# Miniaturization of the Clonogenic Assay Using Confluence Measurement

**DOI:** 10.3390/ijms19030724

**Published:** 2018-03-03

**Authors:** Christian Mayr, Marlena Beyreis, Heidemarie Dobias, Martin Gaisberger, Martin Pichler, Markus Ritter, Martin Jakab, Daniel Neureiter, Tobias Kiesslich

**Affiliations:** 1 Laboratory for Tumour Biology and Experimental Therapies (TREAT), Institute of Physiology and Pathophysiology, Paracelsus Medical University Salzburg, Strubergasse 22, 5020 Salzburg, Austria; christian.mayr@pmu.ac.at (C.M.); marlena.beyreis@pmu.ac.at (M.B.); heidi.dobias@pmu.ac.at (H.D.); 2 Department of Internal Medicine I, Paracelsus Medical University/Salzburger Landeskliniken (SALK), Muellner Hauptstrasse 48, 5020 Salzburg, Austria; 3 Laboratory of Functional and Molecular Membrane Physiology (FMMP), Institute of Physiology and Pathophysiology, Paracelsus Medical University Salzburg, Strubergasse 22, 5020 Salzburg, Austria; martin.jakab@pmu.ac.at; 4 Department for Radon Therapy Research, Ludwig Boltzmann Cluster for Arthritis and Rehabilitation, Institute of Physiology and Pathophysiology, Paracelsus Medical University Salzburg, Strubergasse 22, 5020 Salzburg, Austria; martin.gaisberger@pmu.ac.at (M.G.); markus.ritter@pmu.ac.at (M.R.); 5 Division of Oncology, Department of Internal Medicine, Medical University Graz, Auenbruggerplatz 15, 8036 Graz, Austria; martin.pichler@medunigraz.at; 6 Department of Experimental Therapeutics, The UT MD Anderson Cancer Center, 1515 Holcombe Blvd, Houston, TX 77030, USA; 7 Gastein Research Institute, Institute of Physiology and Pathophysiology, Paracelsus Medical University Salzburg, Strubergasse 22, 5020 Salzburg, Austria; 8 Institute of Pathology, Paracelsus Medical University/Salzburger Landeskliniken (SALK), Muellner Hauptstrasse 48, 5020 Salzburg, Austria; d.neureiter@salk.at; 9 Cancer Cluster Salzburg, Institute of Pathology, Paracelsus Medical University/Salzburger Landeskliniken (SALK), Muellner Hauptstrasse 48, 5020 Salzburg, Austria

**Keywords:** clonogenic assay, clonogenic growth, 96-well microplate format, microplate reader, confluence detection

## Abstract

The clonogenic assay is a widely used method to study the ability of cells to ‘infinitely’ produce progeny and is, therefore, used as a tool in tumor biology to measure tumor-initiating capacity and stem cell status. However, the standard protocol of using 6-well plates has several disadvantages. By miniaturizing the assay to a 96-well microplate format, as well as by utilizing the confluence detection function of a multimode reader, we here describe a new and modified protocol that allows comprehensive experimental setups and a non-endpoint, label-free semi-automatic analysis. Comparison of bright field images with confluence images demonstrated robust and reproducible detection of clones by the confluence detection function. Moreover, time-resolved non-endpoint confluence measurement of the same well showed that semi-automatic analysis was suitable for determining the mean size and colony number. By treating cells with an inhibitor of clonogenic growth (PTC-209), we show that our modified protocol is suitable for comprehensive (broad concentration range, addition of technical replicates) concentration- and time-resolved analysis of the effect of substances or treatments on clonogenic growth. In summary, this protocol represents a time- and cost-effective alternative to the commonly used 6-well protocol (with endpoint staining) and also provides additional information about the kinetics of clonogenic growth.

## 1. Introduction

Since its initial description in the 1950s, the clonogenic assay is an established method to measure the ability of cells to “infinitely” reproduce, that is, generation of progeny or clones [1,2]. For this purpose, cells are seeded at low density for a specific period of time (1–3 weeks). Then, the number of colonies are counted and each colony (i.e., clone) represents a direct progeny of a single, seeded cell that harbored the ability for clonal growth [2,3].

The assay was originally used to study the effects of radiation on cellular survival and growth [4,5]. Today, clonogenic assays are used for a variety of experimental questions, especially in tumor biology [6,7,8,9,10]. Albeit, two-dimensional cell culture assays do not fully recapitulate the physiologic three-dimensional growth of tumor cells, the ability to generate clones is interpreted as a trait of aggressive tumor cells that harbor tumor-initiating capabilities [11,12]. Consequently, the assay is used to test different (primary) cells, cell lines, or cell subpopulations for their “clone-forming” capacity, as well as to test potential anti-tumor substances or treatments for their effects on clonogenic growth [2]. Since stem cells are long-living cells with the potential for ongoing proliferation, determination of clonogenic growth is also used for evaluating the stemness of particular cell populations [5,11,13]. In the context of cancer research, this is especially interesting considering the postulated cancer stem cell (CSC) model. Following this model, a tumor is a hierarchically organized and heterogeneous cell conglomerate with CSCs residing at the top of the hierarchy. Similar to healthy stem cells, CSCs have the ability to self-renew and to generate differentiated cells (tumor bulk) [14]. Because various major clinical problems in cancer management, such as chemoresistance, the formation of secondary tumors, and cancer recurrence, can be explained by the existence of CSCs, assays investigating cancer-stemness, such as the clonogenic assay, are very valuable and widely used [15].

The current standard protocol for the clonogenic assay uses the 6-well or 24-well plate format for cell seeding and staining of colonies (e.g., crystal violet) as an endpoint measurement [2,16]. While frequently used and robust, this procedure has several disadvantages. For instance, the usage of plates with a low number of wells (6-well or 24-well microplate format) does not allow high-throughput analysis of a drug’s effect (including an appropriate number of technical replicates) without excessive and expensive effort of material and time. In addition, information gathered by endpoint analysis is limited as both time-resolved analysis and information about the kinetics of colony growth are not decipherable from an endpoint measurement.

We here describe a modified protocol for the clonogenic assay that miniaturizes the method into a 96-well microplate format, allowing for comprehensive analysis of cells and/or substances on clonogenic growth. Moreover, we describe the utilization of the confluence detection function of a multimode reader as a way for real-time, non-endpoint, and label-free analysis of clonogenic growth. To exemplify the advantages and applicability of our modified protocol, we use PTC-209 as a model substance to inhibit clonogenic growth [6].

## 2. Results

### 2.1. Using the Confluence Measurement to Translate the Clonogenic Assay in a 96-Well Microplate Format

**Figure 1:**
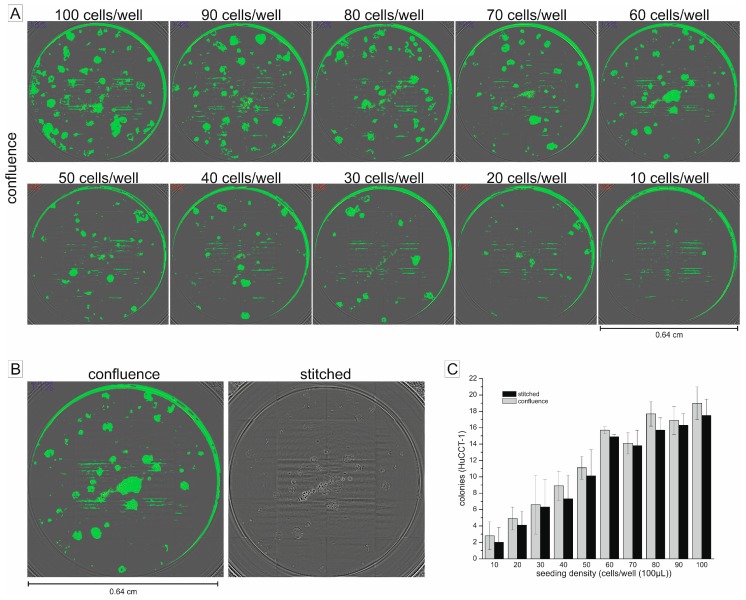
Establishment of cell seeding densities in 96-well microplates and comparison between confluence module detection and stitched pictures. (**A**) Exemplary series of different seeding densities in the 96-well microplate format (100 µL volume per well). Images were taken after 7 days using the confluence module. Green spots represent areas detected as confluent by the confluence module; (**B**) Enlarged exemplary picture pair showing the identical well pictured as a confluence detection photo and a stitched photo, respectively. For better visibility, contrast and brightness were adjusted in the stitched picture; (**C**) Quantification of the number of colonies after 7 days using different cell seeding densities (*n* ≥ 3).

To test whether confluence determination in a multimode reader is suitable for detecting clonogenic growth, as well as to translate the clonogenic assay into the more practicable 96-well microplate format, we performed experiments that allowed simultaneous processing of both of these questions. As seen in Figure 1A, we used a cell dilution series ranging from 10 to 100 cells per well to test the accuracy of the confluence function to identify cell colonies as continuous confluent areas. Areas that were identified as confluent were highlighted in green by the multimode reader software. For each well, we additionally took a conventional bright-field image covering the whole well (“stitched” imaging option in the multimode reader software). We next compared the amount and the position of the colonies displayed in the stitched images with the areas detected as confluent by the confluence detection function. As shown in Figure 1A, confluence measurement was able to accurately detect clonogenic growth over the tested cell seeding density range. Moreover, a comparison of the stitched images with images of the confluence detection function clearly demonstrates that visible clones in the stitched images were detected and highlighted by the confluence measurement (exemplified in Figure 1B). As quantified in Figure 1C, detection of colonies by the confluence measurement was similar to the results obtained from conventional stitched images and robust for various cell seeding densities and experimental series.

For optimization of the cell seeding density, we quantified the number of colonies grown after 7 days (longer time periods resulted in excessive colony merging) for each seeding concentration. We observed that a seeding density of 60 cells per well (complying a concentration of 600 cells/mL) led to reproducible and plausible results (Figure 1C). Combined with the observation that after 7 days, a seeding density of 60 cells per well showed no excessive merging of single colonies (Figure 1A,B), we chose this seeding density for further experiments.

### 2.2. Non-Endpoint Analysis of Clonogenic Growth in the 96-Well Format

**Figure 2:**
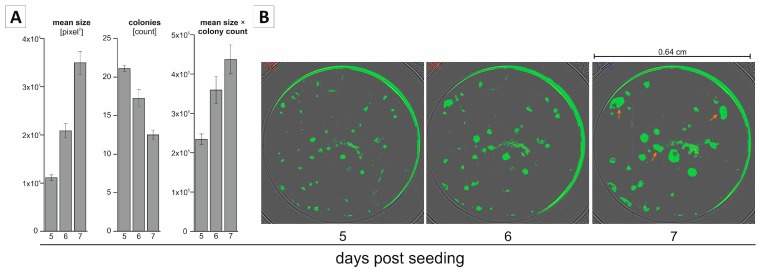
Evaluation of clonogenic growth in the 96-well format over time using the confluence detection. (**A**) Quantification of clonogenic growth at different time points (*n* ≥ 3); (**B**) Exemplary pictures of the identical well 5, 6, and 7 days post seeding, using the confluence detection function. Green spots represent areas detected as confluent by the confluence module. Orange arrows indicate merged colonies.

Analysis of the classical 6-well clonogenic assay is based on staining of colonies and an endpoint measurement. The confluence function of a multimode reader allows non-endpoint analysis of clonogenic growth and the gathering of additional information about the kinetics of clonogenic growth, that is, the determination of colony size and colony count in one well over defined time periods. To test and demonstrate the robustness of non-endpoint analysis of clonogenic growth in the 96-well format using the confluence measurement function, we measured the mean size and number of colonies at 5, 6, and 7 days post seeding. As expected, we observed a reproducible increase in the mean colony size over time (Figure 2A, left bar chart). This is also illustrated in the exemplary confluence images in Figure 2B, showing a steady growth of the individual colonies (pictures of the same well at different time points). Interestingly, we saw a decline in the number of colonies over time, which at first glance might look contradictory (Figure 2A, middle bar chart). This phenomenon can be explained by looking at the confluence images shown in Figure 2B. Due to the increase in size and a potential initial spatial proximity, two (or more) individual colonies may merge, meaning that the overall colony count will decrease. Examples of such colony merging are marked in Figure 2B (orange arrows). We additionally interpreted the data of our non-endpoint measurement by multiplying the mean size and colony count to integrate both parameters (size and number) in our analysis. This factor increases over time (Figure 2A, right bar chart) and reflects a similar kinetic growth pattern as seen for mean size (Figure 2A, left bar chart).

### 2.3. Application of Non-Endpoint Measurement—Dose-Dependent Effect of PTC-209 on Clonogenic Growth

**Figure 3:**
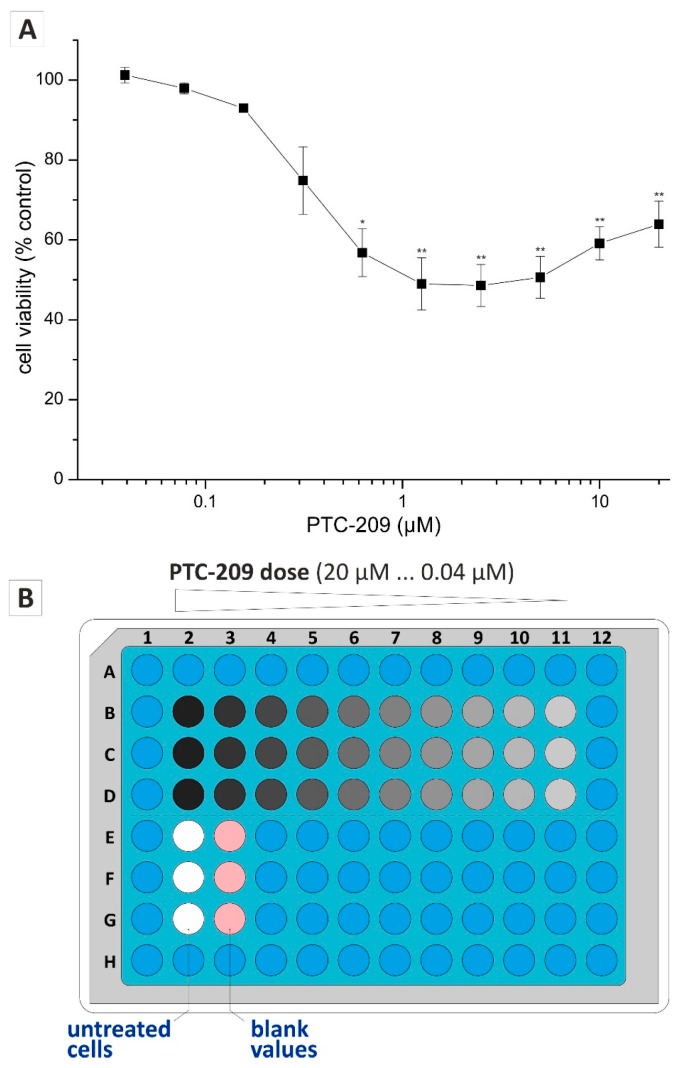
Setup for dilution series experiments for the clonogenic assay in the 96-well format. (**A**) Concentration-dependent effect of PTC-209 on cell viability of HuCCT-1 cells after 72 h (mean of *n* ≥ 3 experiments); (**B**) Blue-colored and light blue-colored wells and plate areas, respectively, indicate that medium has been added to avoid evaporation during long-term incubation. Using this layout, 60 cells per well were seeded 24 h prior to the addition of a PTC-209 dilution series. * *p* < 0.05; ** *p* < 0.01.

In most experimental setups using the clonogenic assay, the effect of a specific substance or treatment is investigated. Implementation of the demonstrated miniaturization of the clonogenic assay in the practicable 96-well format, as well as the non-endpoint analysis using confluence determination of a multimode reader, allows time- and cost-effective screening of a substance regarding a concentration-dependent effect on clonogenic growth. To verify these statements, we used PTC-209 (BMI1 Polycomb Ring Finger Oncogene (BMI1) inhibitor) that we recently demonstrated to inhibit clonogenic growth in biliary tract cancer cells [6]. Beforehand, we performed a classical dose-response curve of PTC-209 to determine the concentration range for the subsequent clonogenic assay experiments. Based on the evaluation of the cytotoxic effect of PTC-209, we selected a dilution series ranging from 0.04 to 20 µM (Figure 3A).

**Figure 4:**
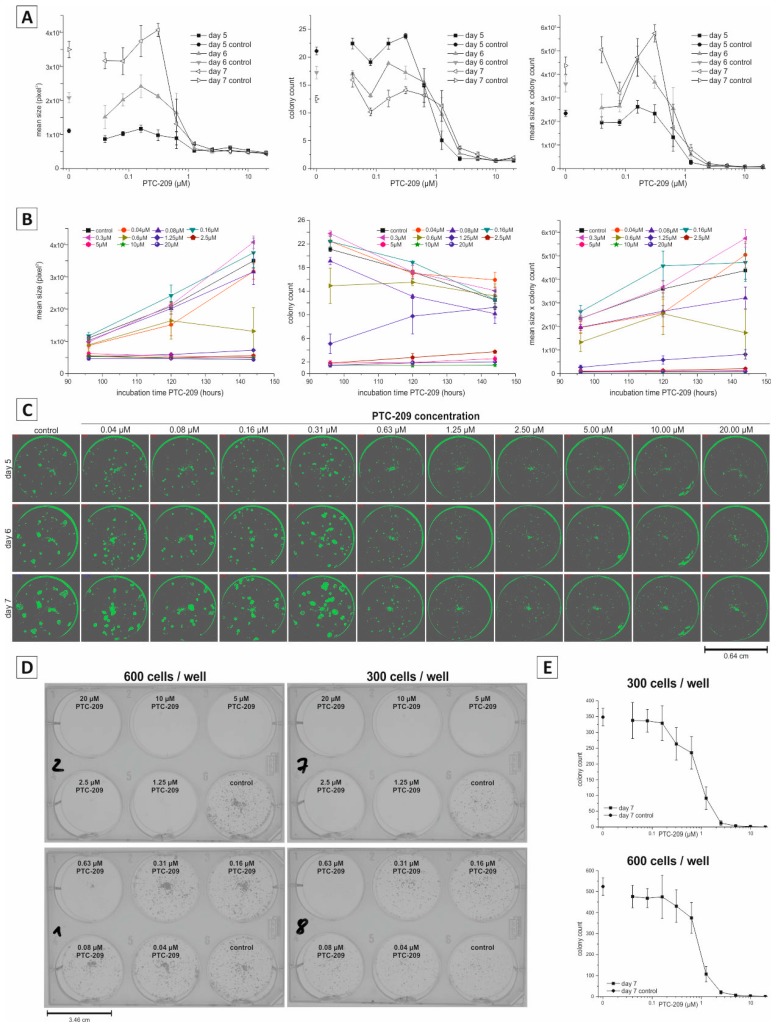
Evaluation of the inhibitory effect of PTC-209 on clonogenic growth in the 96-well format using non-endpoint confluence measurement. (**A**) Dose-dependent effect of PTC-209 on mean colony size, number of colonies, and the factor mean size × colony count after 5, 6, and 7 days of incubation, respectively. Data are given as mean values ± standard error of mean; (**B**) Time-resolved analysis of the effect of different PTC-209 concentrations on mean size, number of colonies, and the factor mean size × colony count. Data are given as mean values ± standard error of mean; (**C**) Representative confluence images for a complete experimental series. From left to right: dose-dependent effect of PTC-209 on clonogenic growth. From top to bottom: time-resolved effect of various PTC-209 concentrations in the same well; (**D**) Exemplary images of a clonogenic assay performed in the 6-well plate format taken with a digital camera following crystal violet staining; (**E**) Dose-dependent effect of PTC-209 on colony count after 7 days in the 6-well format. Data are given as the mean values ± standard error of mean. All data were obtained from *n* ≥ 3 individual experiments.

Figure 3B illustrates the 96-well microplate layout for clonogenic growth analysis. Using ten different drug concentrations and technical triplicates, this experimental setup and layout allows a comprehensive dose-response analysis to give detailed information on concentration-dependent effects of a specific substance or treatment. The effect of PTC-209 on clonogenic growth was measured at 5, 6, and 7 days post-incubation, using the confluence determination of the multimode reader followed by semi-automatic image analysis. As expected, PTC-209 reduced the mean size of the colonies in a concentration-dependent manner (Figure 4A). This effect was reproducible for each time point, meaning, that despite the overall increase in mean colony size over time for the untreated and non-inhibitory PTC-209 samples, the inhibitory PTC-209 concentration remained constant at about 0.63 µM (Figure 4A, left graph). Similarly, PTC-209 reduced the colony count in a concentration-dependent manner. However, as noticed and described before, the overall colony count decreased over time for untreated and non-inhibitory PTC-209 concentrations due to the merging of individual colonies (Figure 4A, middle graph). As expected, when multiplying mean size and colony count, we observed a concentration-dependent decline at PTC-209 concentrations that showed an inhibitory effect on either mean size only or colony count only (Figure 4A, right graph, for statistics see supplementary Table S1). Additionally, we analyzed the effect of all PTC-209 concentrations used in our dilution series on clonogenic growth over time (Figure 4B). By doing this we could clearly determine that for mean size, the lowest concentration that showed a considerable inhibitory effect was 0.63 µM (Figure 4B, left graph), whereas for colony count only PTC-209 concentrations ≥2.5 µM considerably reduced the number of colonies (Figure 4B, middle graph). Time-resolved analysis of the factor mean size × colony count indicated that 0.63 µM was the lowest PTC-209 concentration with substantial inhibitory effects on clonogenic growth (Figure 4B, right graph). Figure 4C shows the confluence detection images of a complete experimental series (exemplary). From top to bottom, confluence images of the same well at different time points are shown, whereas from left to right, confluence images of samples treated with different PTC-209 concentrations for the same time point are presented. These pictures clearly illustrate the observations quantified in Figure 4A,B: for control and non-inhibitory PTC-209 concentrations (0.04–0.31 µM), a clear increase in clonogenic growth over time is visible; however, inhibitory PTC-209 concentrations ≥0.63 µM diminished or abolished clonogenic growth at all time points.

To exclude format-specific effects, we additionally performed PTC-209 dilution series experiments in the 6-well microplate format (endpoint analysis after 7 days of PTC-209 incubation). As illustrated in Figure 4D,E, non-inhibitory (ranging from 0.04–0.31 µM) and inhibitory (≥0.63 µM) PTC-209 concentrations in the 6-well format were similar to the 96-well format. This demonstrates that the results we generated using our newly developed protocol are independent of the dimensions of the cell culture receptacle used.

## 3. Discussion

Clonogenic assays are widely used in the field of cancer research for the evaluation of cancer stemness, cellular growth, and the cytotoxic effects of radiation or substances on cancer cells [2,4,5,15,17]. Clonogenic growth—that is, the measurement of the ability of cells to generate clonal progeny at low cell densities—serves as a surrogate marker for the tumor-initiating capacity of cancer cells and, therefore, provides important information in addition to other established cytotoxic or CSC-characterizing assays and protocols. For example, cells positive for the CSC marker aldehyde dehydrogenase 1 were shown to harbor a significantly enhanced capability to form clones compared to aldehyde dehydrogenase 1-negative cells [17,18,19]. Three-dimensional systems and assays such as the sphere assay are commonly used and provide additional information beyond two-dimensional systems, as they better represent the physiologic situation in the tumor [20]. Information gathered by the clonogenic assay can and should therefore be complemented by data from three-dimensional assays. The intrinsic characteristics of cells to generate clones in two-dimensional systems most likely also allows these cells to form spheres in a three-dimensional setting. Sun and coworkers have shown that the same specific subset of endometrial cancer cells displaying CSC surface markers were able to form both, numerous spheres and numerous colonies compared to the other cellular populations investigated [9]. Moreover, we were recently able to demonstrate that PTC-209 not only abolished clonogenic growth in BTC cells but also reduced sphere formation potential [6]. The currently used protocol for the clonogenic assay is based on endpoint analysis (via staining e.g., with crystal violet) in the 6-well plate format [2,16]. Here, we developed a new and practicable protocol by miniaturizing the assay into a 96-well microplate format and by using label-free and non-endpoint analysis by means of confluence detection in a multimode microplate reader. After appropriate optimization of seeding density, this protocol is suitable for cell lines that form colonies and grow in the 96-well format in a defined two-dimensional layer.

Our protocol has several advantages over the commonly used 6-well protocol with endpoint analysis. First, using a 96-well format allows a more flexible and detailed investigation of clonogenic growth as the higher number of wells allows direct comparison of several cell seeding numbers, treatment periods, and/or drug dilutions. By this, multi-parametric screening of the effects of diverse substances or diverse concentration ranges of a single substance on clonogenic growth becomes technically more feasible. Second, usage of the 96-well format allows meaningful inclusion of technical replicates, blank values, and solvent controls. Third, miniaturization of the clonogenic assay in the 96-well format is both time and cost efficient as more samples and/or treatments (including technical replicates) can be handled on one plate and lower volumes (e.g., media and drugs) are needed. This is especially interesting for treatments that are expensive or the availability of the particular treatment itself is a limiting factor (e.g., drug-based, siRNA-based, CRISPR/Cas9-based). Fourth, there is a tendency of cells and colonies to clump and concentrate in the well’s center in the 6-well plate format, making analysis difficult and inaccurate. Throughout our experiments, we did not observe such aggregation in the 96-well format. Fifth, a major advantage of our protocol is the possibility to continuously analyze clonogenic growth. We were clearly able to demonstrate that confluence detection in a 96-well microplate format presents a suitable format for continuous monitoring and analysis of clonogenic growth. In addition, high-throughput analysis for comprehensive drug screening combines well with the miniaturized 96-well protocol using automated confluence detection for efficient and reliable evaluation of clonogenic growth. Utilization of the confluence detection function as a readout of clonogenic growth not only allows practicable analysis but also the generation of new scientific information. First and foremost, by combining the 96-well format with confluence imaging, there is no need for endpoint colony staining as the readout. Instead, non-endpoint, live evaluation at different time points is possible, allowing for time- and cost-efficient time course experiments for the evaluation of clonogenic growth. Compared to simple endpoint analysis experiments, such time course investigations can give information about the time-resolved effects of a specific substance or treatment on kinetics of clonogenic growth. Besides that, the procedure obviates handling with harmful staining solutions (such as crystal violet). For clonogenic assays, it is essential to perform the experiments in a proper timeframe to avoid excessive merging of colonies. Utilizing non-endpoint confluence detection as the readout allows the measurement of clonogenic growth at different time points to optimize these (cell-specific) measurement time ranges without the need to set up different plates. Moreover, provided the necessary functionality of a multimode reader (temperature and gas control, anti-evaporation cassette), our protocol enables continuous real-time measurement of clonogenic growth over hours or days, dependent on the individual experimental setup and scientific questioning. This is not only interesting for continuous monitoring of clonogenic growth but also for cell line-specific beforehand optimization of the experimental setup.

## 4. Materials and Methods

### 4.1. Cell Culture and Substances

PTC-209 was obtained from Selleckchem (Houston, Texas, USA), dissolved in dimethyl sulfoxide (DMSO; Sigma Aldrich/Merck, Vienna, Austria) at a stock concentration of 10 mM and stored in aliquots at −20 °C. Resazurin was obtained from Sigma Aldrich/Merck (Vienna, Austria) and dissolved in Dulbecco’s Phosphate Buffered Saline (DPBS; Biochrom, Berlin, Germany). For all experiments, the bile duct carcinoma cell line HuCCT-1 was used (obtained from JCRB; [21]). HuCCT-1 cells were cultured in high glucose Dulbecco’s modified Eagle’s medium (DMEM; Gibco, ThermoFisher Scientific, Vienna, Austria) supplemented with 10% (*v*/*v*) fetal bovine serum (FBS; Gibco, ThermoFisher Scientific, Vienna, Austria) as described elsewhere [22].

### 4.2. Clonogenic Assay

Cell seeding density in the 96-well microplate format (Starlab, Hamburg, Germany) was optimized for HuCCT-1 cells in a cell seeding dilution series (range 100–1000 cells/mL, which equals 10 to 100 cells per 96-well seeded at 100 µL per well). Colonies were grown in a humidified cell culture incubator (37 °C, 5% CO_2_) and counted after 7 days using the confluence detection function of a Spark^®^ multimode reader (Tecan, Groedig, Austria). For appropriate measurement of confluence, we chose the following setup in the multimode reader’s control software (Spark Control™, V2.2, Tecan): 96-well flat transparent with lid and whole well confluence detection. Comparison of confluence pictures with stitched images was performed to evaluate appropriate detection of colonies by the confluence measurement function.

The cytotoxic effect of PTC-209 on clonogenic growth was evaluated by seeding 600 cells/mL in the 96-well format (100 µL seeding volume; equals 60 cells per well). To avoid evaporation, remaining wells and plate interstices were filled with culture media. Cells were incubated overnight to allow attachment to the well bottom. After washing carefully with culture media, cells were incubated with a dilution series of PTC-209 (range 0.04–20 µM) and confluence was measured after 5, 6, and 7 days using the Spark Control software settings as described above (numbers at the left upper corner of the confluence pictures indicate % confluent area of the respective well). ImageJ (V1.48, NIH, Bethesda, MD, USA) was used for counting and measurement of colony mean size as described earlier [16]. In brief, after converting the image to 8-bit format, the threshold was adjusted to reduce non-specific background signal. Then, colonies were counted and measured using the “Analyze Particles” function choosing appropriate size (≥3500 square pixels for HuCCT-1 cells) and circularity (≥0.5) settings based on sample images. Experiments were performed in technical triplicates and biological quadruplicates.

For evaluation of the effect of PTC-209 on clonogenic growth in the 6-well plate format, we used the same cell concentration (600 cells/mL; 3 mL seeding volume, equals 1800 cells per well) and the same protocol as described for the 96-well format. We performed an additional experimental series with a seeding density of 300 cells/mL (equals 900 cells per well), as 600 cells/mL resulted in enhanced colony merging for untreated cells and non-inhibitory PTC-209 concentrations. After 7 days, colonies were stained with a staining solution (80% PBS, 20% methanol (Sigma Aldrich, Vienna, Austria), 0.1% crystal violet (Sigma Aldrich)) for 20 min, carefully washed with PBS, air-dried, documented with an Om-D E-M10 Mark II digital camera (Olympus, Vienna, Austria), and manually quantified using ImageJ (V1.48).

### 4.3. Drug Cytotoxicity

The dose-dependent cytotoxic effect of PTC-209 was investigated on cells grown in 96-well microplates using a dilution series (range 0.04–20 µM) and an incubation time of 72 h in serum-free DMEM to avoid interactions of the substance with components of the serum. Quantification of cell viability was done using the resazurin assay and the Spark multimode reader (Tecan) as described [22].

### 4.4. Statistics

All data points represent mean values of at least three biological replicates ± standard error of the mean. Paired Student’s t-test was used for calculation of significance between groups. Statistical results were considered significant (*) or highly significant (**) at *p* < 0.05 and *p* < 0.01, respectively. Calculations were done with OriginPro 2017 (V8, OriginLab, Northampton, MA, USA).

## 5. Conclusions

In summary, we demonstrated that miniaturization of the clonogenic assay into a 96-well format combined with the utilization of confluence determination on a multimode reader serves as a cost- and time-effective protocol to measure 2D clonogenic growth. Moreover, this protocol allows for meaningful time-resolved and continuous measurement, as well as for comprehensive dilution series-based evaluation of the effect of substances or treatments on clonogenic growth.

## Supplementary Materials

Supplementary materials can be found at http://www.mdpi.com/1422-0067/19/3/724/s1.
