# Supplementary material for: Miniaturization of the Clonogenic Assay Using Confluence Measurement
Source: Int J Mol Sci. 2018 Mar 3;19(3):724. doi: 10.3390/ijms19030724 (PMC5877585; doi:10.3390/ijms19030724)
Supplement: Supplementary file 1 [file ijms-19-00724-s001.pdf]

Mayr et al.

Supplementary Table 1 **Miniaturization of the clonogenic assay using confluence measurement**

Day	Parameter	PTC-209 (µM)									
		**20.00**	**10.00**	**5.00**	**2.50**	**1.25**	**0.63**	**0.31**	**0.16**	**0.08**	**0.04**
**5**	Mean size	**	*	**	**	**					
	Colony count	**	**	**	**	**					
**6**	Mean size	**	**	**	**	**					
	Colony count	**	**	**	**						
**7**	Mean size	**	**	**	**	**				*	
	Colony count	**	**	**	**						
